# Echocardiographic Alterations in Subjects with Sarcopenia and Right Heart Failure

**DOI:** 10.3390/medsci13040313

**Published:** 2025-12-10

**Authors:** Arturo Orea-Tejeda, Luis Aldo Delgado-Pérez, Benigno Valderrábano-Salas, Dulce González-Islas, Álvaro Montañez-Orozco, José Carlos Ruan-Díaz, María José Hernández-Hernández, Edgar Lozano-Hernández, Carlos Patricio Chávez-Guzmán, Karla García-Díaz

**Affiliations:** Cardiology Service, Instituto Nacional de Enfermedades Respiratorias “Ismael Cosío Villegas”, Mexico City 14080, Mexico; oreatart@gmail.com (A.O.-T.); luis.delgadoperez@viep.com.mx (L.A.D.-P.); mdvalderrabano@gmail.com (B.V.-S.); alvaro.dox@gmail.com (Á.M.-O.); ruancardio@gmail.com (J.C.R.-D.); majohdz99xd@gmail.com (M.J.H.-H.); edgar.lozano.hdez23@gmail.com (E.L.-H.); ccarlosppatrik@gmail.com (C.P.C.-G.); karlayailingarciadiaz@gmail.com (K.G.-D.)

**Keywords:** sarcopenia, right heart failure, muscle wasting, left ventricular diastolic dysfunction

## Abstract

Background: Chronic heart failure (HF) is a significant public health issue. The principal risk factors for left ventricular diastolic dysfunction (LVDD) include older age, female sex, obesity, hypertension, smoking, and diabetes, among others, all of which can reduce physical activity. Additionally, peripheral factors such as skeletal muscle mass (SMM) abnormalities decrease maximal oxygen consumption. In elderly HF patients, the prevalence of sarcopenia is higher than in those without HF; however, the relationship between sarcopenia and HF remains insufficiently explained, particularly in right HF (RHF). Our objective was to describe the echocardiographic alterations between sarcopenic and non-sarcopenic subjects with RHF. Methods: A cross-sectional study was conducted. Outpatients aged 18 years or older with a confirmed diagnosis of RHF were included. Sarcopenia was defined according to EWGSOP2. Results: A total of 183 patients were included; 24.5% had sarcopenia. The mean age was 64.34 ± 13.97 years. Echocardiographic characteristics revealed evidence of LVDD in sarcopenic subjects, as indicated by lower E wave velocity, E/A ratio, and e’ lateral and medial values, as well as lower right ventricular (RV) wall thickness compared with non-sarcopenic subjects. The multivariate model showed that sarcopenia subjects had lower RV wall thickness (B: −1.36 mm, 95% CI: −2.30 to −0.42), e’ medial (B: −1 cm/s, 95% CI: −1.99 to −0.02), and e’ lateral (B: −1.78 cm/s, 95% CI: −2.97 to −0.60). Conclusions: The prevalence of sarcopenia in RHF patients was 24.6%, which was associated with LVDD and lower RV wall thickness, suggesting a loss of cardiac muscle mass.

## 1. Introduction

Heart failure (HF) is a significant public health problem [1]. In its chronic form, that is chronic HF (CHF), it increases with age, occurring in similar proportions in both reduced ejection fraction (HFrEF) and preserved ejection fraction (HFpEF), both of which carry a poor prognosis [2]. Patients with preserved HFpEF primarily report symptoms of dyspnea or early fatigue [3]. This syndrome is highly prevalent among older individuals and is characterized by clinical symptoms or signs with a left ventricular ejection fraction (LVEF) > 50% and increased cardiac filling pressures. HFpEF is also associated with increased morbidity and mortality, leading to substantial medical and economic burdens [4]. Diastolic dysfunction plays a central role in the principal pathogenic and asymptomatic form of left ventricular diastolic dysfunction (LVDD). It represents an important predictor of both fatal and non-fatal cardiovascular events, often progressing to symptomatic HF [5]. The principal risk factors for LVDD include older age, female sex, obesity, hypertension, smoking, diabetes mellitus, coronary artery disease, valvular heart disease, atrial fibrillation, and chronic obstructive pulmonary disease (COPD) [6]. However, peripheral factors such as skeletal muscle abnormalities may decrease maximal oxygen consumption and explain improvements observed after exercise training [7]. Muscle mass declines by 1–2% annually [8], while muscle strength decreases by approximately 1.5%; this decline accelerates to approximately 3% per year after age 60 [9].

On the other hand, sarcopenia is a progressive and generalized skeletal muscle disorder characterized by low muscle strength and low skeletal muscle mass (SMM) that increases the risk of falls, fractures, physical disability, and mortality [10].

Sarcopenia has been associated with various cardiovascular diseases, particularly HF [11]. The prevalence of sarcopenia is 31%, and the incidence of sarcopenia in elderly HF patients is higher than in those without HF [12].

HF and sarcopenia share interconnected pathophysiological mechanisms, including chronic inflammation, hormonal changes, endothelial dysfunction, insulin resistance, reduced muscle blood flow, impaired muscular oxygen uptake, mitochondrial dysfunction, and oxidative stress, which perpetuate a vicious cycle [13,14].

A previous study shows that subjects with LVDD have a higher prevalence of sarcopenia compared to subjects without LVDD [15]. In addition, in a cross-sectional study by Jung et al., it was observed that subjects with sarcopenia had a higher risk of LVDD, and this risk increased in subjects with sarcopenic obesity [16]. However, in both studies, RV echocardiographic parameters were not evaluated.

As both skeletal muscle and myocardium are striated muscles, we hypothesized that their size may be affected by skeletal muscle loss, which can reduce cardiac muscle mass and function. However, the association between sarcopenia and HF remains poorly understood, particularly in the context of RHF. Our objective was to describe the echocardiographic alterations between sarcopenic and non-sarcopenic subjects with RHF.

## 2. Materials and Methods

### 2.1. Study Design and Population

A cross-sectional study was conducted at the Instituto Nacional de Enfermedades Respiratorias Ismael Cosío Villegas in Mexico City, Mexico, from 1 August 2017, to 31 January 2025. Outpatients over 18 years with a confirmed diagnosis of RHF based on the right ventricular diameter (RVD) criteria from the European Society of Cardiology [17,18] were included. Exclusion criteria included subjects with cancer, human immunodeficiency virus, neurological disease, or a history of hospitalization within the previous three months.

The study was conducted in accordance with the Declaration of Helsinki and was approved by the Research and Ethics Committees of the Instituto Nacional de Enfermedades Respiratorias “Ismael Cosío Villegas” (Project Approval Number E02-18, 26 February 2017). Informed consent was obtained from all subjects involved in the study.

### 2.2. Outcome Measures

Echocardiographic parameters, body composition, clinical, and demographic variables were evaluated as part of the clinical management of patients at our institute.

### 2.3. Right Heart Failure (RHF)

Two-dimensional echocardiography was performed using a General Electric Vivid E95 echocardiography machine (GE ultrasound System, Horten, Norway) by trained echocardiographers. RHF was defined by a tricuspid annular plane systolic excursion < 17 mm, fractional area change < 35%, or tissue-Doppler-derived image S’ < 9.5 cm/s.

The left ventricle diameter (LVD), left atrial diameter, RVD, right atrial diameter, aorta diameter, pulmonary artery diameter (PA), interventricular septum diastolic thickness (IVSD), left ventricular posterior wall diastolic thickness (LVPWD), LVEF, and left ventricular fractional shortening were measured. Left ventricular mass (LVM) was calculated using the equation: LVM (g) = 0.8 × 1.04 × [(LVD + IVSD + LVPWD)^3^ − LVD^3^] + 0.6.

### 2.4. Handgrip Strength

Handgrip strength was measured using a mechanical Smedley Hand Dynamometer (Stoelting, Wood Dale, UK) according to the technique described by Rodriguez et al. [19].

### 2.5. Bioelectrical Impedance Analysis

Total body composition was measured using whole-body bioelectrical impedance analysis with four-pole multifrequency equipment (BodyStat QuadScan 4000, BodyStat Ldt, Isle of Man, UK) following standard technique [20]. The same operator conducted the measurements in the morning in a comfortable area free of drafts, with portable electric heaters. The area was cleaned before the study. Subjects were fasting and had not exercised for eight hours prior or consumed alcohol for 12 h before the study. During the study, participants were supine, with arms separated from the trunk by approximately 30° and legs by approximately 45°. Electrodes were placed on the hand and the ipsilateral foot.

### 2.6. Appendicular Skeletal Muscle Mass Index (ASMMI)

ASMMI was assessed using Sergi’s equation [21]: ASMMI (kg/m^2^) = [−3.964 + (0.227 × (Height^2^ (cm)/R) + (0.095 × Weight + (1.384 × Sex) + (0.064 × Xc)/Height (m^2^)].

### 2.7. Sarcopenia

Sarcopenia was defined according to EWGSOP2 [10] as the presence of low muscle strength (handgrip strength < 27 kg for men and <16 kg for women) and low muscle mass (ASMMI < 7.0 kg/m^2^ for men and <6.0 kg/m^2^ for women).

### 2.8. Statistical Analysis

Analyses were performed using STATA version 14 (Stata Corp., College Station, TX, USA). Categorical variables were expressed as frequencies and percentages. The Shapiro–Wilk test assessed the normality of continuous variables; normal variables were expressed as mean and standard deviation, while non-normal variables were reported as median and percentiles 25–75. Comparisons between study groups (sarcopenia vs. non-sarcopenia) were analyzed using the chi-square test for categorical variables and the t-student test or Mann–Whitney U test for continuous variables.

A linear regression test was performed to assess the association between sarcopenia and echocardiographic parameters. The model was adjusted using bivariate analysis for variables with *p*-values < 0.05. Results were reported as Beta (β) with 95% confidence intervals (95% CI). A *p*-value < 0.05 was considered statistically significant.

## 3. Results

One hundred and eighty-three patients were included, all diagnosed with RHF, and sarcopenia was identified in 45 (24.5%). The mean age was 64.34 ± 13.97 years; sarcopenic subjects were older (74.44 ± 10.19 vs. 61.05 ± 13.48 years, *p* < 0.001), had a higher prevalence of men (73.33% vs. 55.80%, *p* = 0.037), and had a higher prevalence of COPD (51.11% vs. 31.16%, *p* < 0.016). They also had lower serum albumin levels, although this was not statistically significant compared to non-sarcopenic subjects (Table 1).

**Figure 1:**
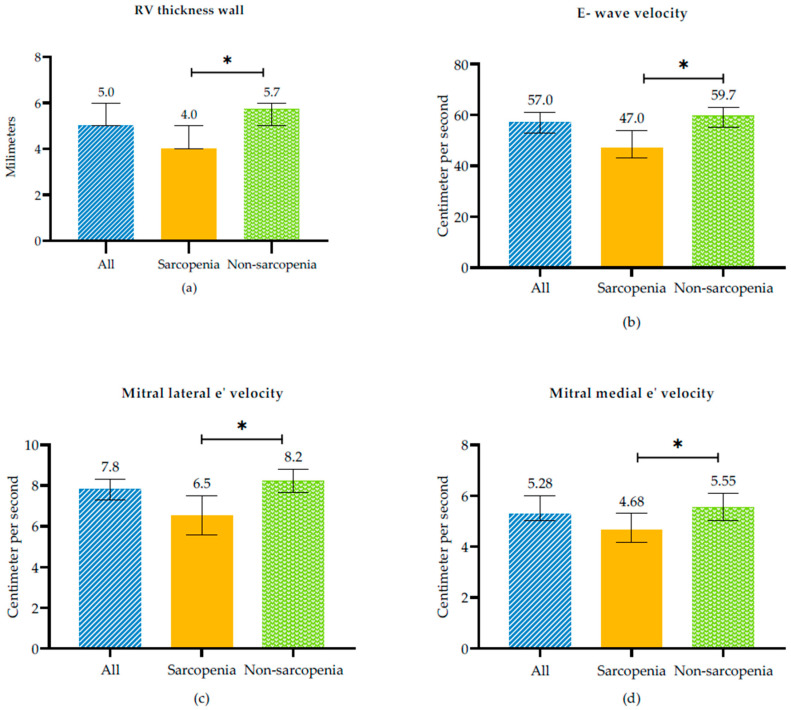
Echocardiographic variables between sarcopenic and non-sarcopenic subjects in RHF. (**a**) RV Thickness wall, (**b**) E-wave velocity, (**c**) Mitral lateral e’velocity and (**d**) Mitral medial velocity. * *p*-value < 0.005 between sarcopenia and non sarcopenia.

In terms of echocardiographic characteristics, in sarcopenic subjects was observed evidence of LVDD expressed as indicated by reduced E wave velocity (47 [35–64.5] vs. 59.7 [49–76], *p* = 0.006), E/A ratio (0.60 [0.51–0.87] vs. 0.76 [0.63–0.97], *p* = 0.005), and mitral lateral e’velocity (6.53 ± 2.88 vs. 8.22 ± 3.04, *p* = 0.003) and mitral medial e’velocity (4.68 [3.8–5.87] vs. 5.55 [4–7.62], *p* = 0.022) values compared with non-sarcopenic subjects In addition, sarcopenic subjects had lower RV thickness wall (4 [3.81–5.15] vs. 5.73 [4.95–7], *p* = 0.002) than non-sarcopenic subjects (Figure 1, and Table 2).

Table 3 presents the linear regression model adjusted for sex, age, and COPD, which showed that subjects with sarcopenia have 1.36 mm lower RV wall thickness (β: −1.36 mm, 95% CI: −2.30 to −0.42), 1 cm/s lower mitral medial e’velocity (β: −1 cm/s, 95% CI: −1.99 to −0.02), and 1.78 cm/s lower mitral lateral e’velocity (β: −1.78 cm/s, 95% CI: −2.97 to −0.60) than non-sarcopenic subjects. In addition, a statistical trend was observed: sarcopenic subjects had a lower LVEF (β: −5.33%, 95% CI: −11.35 to 0.68), as well higher right atrial index (β: 8.57 mL/m^2^, 95% CI: −0.04 to 17.20), and mid-cavity RV diameter (β: 0.24 cm, 95% CI: −0.02 to 0.52) than non-sarcopenic patients.

## 4. Discussion

The main finding of this study was the demonstration of echocardiographic alterations, specifically the LVDD, in subjects with sarcopenia and RHF. Similar results were found in HFpEF subjects by Bekfani et al. They observed that subjects with an E/e’ ratio > 15 had lower muscle strength and appendicular muscle mass than those with an E/e’ ratio < 8 [22]. Decreased compliance and impaired relaxation, along with increased left ventricular end-diastolic pressure, characterize diastolic dysfunction as a central factor in the pathogenesis of HFpEF [23]. In asymptomatic individuals, LVDD has an important role as a predictor of both fatal and non-fatal cardiovascular events and CHF [3], resulting from several factors, including older age, female sex, obesity, hypertension, smoking, diabetes mellitus, coronary artery disease, valvular heart disease, atrial fibrillation, and COPD [23], in addition to these factors, and despite high pulmonary pressures. The consequence of this RV dysfunction is a severe hemodynamic impact due to ventricular interdependence, retrograde congestion toward the venous system, and an anterograde reduction in cardiac output to the pulmonary and systemic circulations.

All of these factors are identified as precursors of CHF, whose prevalence increases with age and occurs in similar proportions in those with HFrEF or HFpEF, both of which carry a poor prognosis [2]. In addition to these factors, despite RV pressure overload due to pulmonary pathology and increased LV filling pressure, the RV wall thickness is reduced.

Several studies, including the Korea National Health and Nutrition Examination Survey, have reported an association between sarcopenia and an increase in cardiovascular diseases. Additionally, it has been linked to HF patients who develop secondary sarcopenia [24]. The incidence of sarcopenia in elderly HF patients is higher than in patients without HF [12]; however, the relationship between sarcopenia and HF remains unclear.

Sarcopenia is typically secondary to reduced exercise capacity in HF, but it is also possible that in primary sarcopenia, cardiac dysfunction may already be present [25]. Furthermore, metabolic changes associated with obesity, a risk factor for cardiovascular diseases, may also affect muscle function through fat infiltration of skeletal muscle (sarcopenic obesity), as adipocytes increase cytokine release [26]. Moreover, the progression of sarcopenia has been associated with increased production of pro-inflammatory cytokines, inadequate nutrition (particularly protein), cellular apoptosis, and mitochondrial dysfunction [27]. Other factors that deteriorate functional capacity include insulin resistance, metabolic syndrome, and lack of exercise [28].

Conversely, heart disease may induce sarcopenia through inflammation, insulin-like growth factor-1, angiotensin, sex hormones, myostatin, physical inactivity, and malnutrition [29]. Some authors have found that LVM was positively correlated with SMM and not associated with LVEF or shortening fraction, supporting the hypothesis that sarcopenia is related to cardiac atrophy as an aging change and also affects myocardial tissue. This suggests that the reduction in LVM may represent myocyte loss that occurs alongside SMM loss as a systemic manifestation, explaining the primary symptom in patients with HFpEF: exercise intolerance [25,26].

The role of peripheral factors, such as SMM, strength, and function, remains poorly understood. In a multicenter European study involving symptomatic stable HFpEF outpatients, 19.7% presented with sarcopenia, indicating muscle wasting [22]. These patients exhibited reduced exercise capacity, as measured by cardiopulmonary exercise tests and six-minute walk tests. Furthermore, several chronic diseases characterized by systemic inflammation, such as COPD, can lead to sarcopenia. One study found that 14.5% of patients with COPD had sarcopenia, with its prevalence increasing with COPD status [30].

Fülster et al. reported significantly elevated serum IL-6 levels in CHF patients with muscle wasting [7]. In our study, participants with sarcopenia also exhibited reduced RV wall thickness (*p* < 0.01; Figure 1 and Table 2). SMM was strongly and positively associated with LVM. In our study, we also observed, in the multivariate model after adjusting for gender, age, and COPD, a reduced RV wall thickness, as noted by other authors in patients with sarcopenia and in symptomatic stable HFpEF outpatients [22]. We did not find significant differences in left ventricular parameters between sarcopenic and non-sarcopenic patients. However, Zhang et al. found that sarcopenia was independently associated with lower LVM after adjusting for diabetes and hypertension. They also described higher body mass index, lower handgrip strength, and a smaller left atrium. Among the 22.8% of patients hospitalized for cardiovascular diseases and cardiovascular-related deaths, nonfatal myocardial infarction, and stroke, patients with sarcopenia also had a smaller RV size [31]. As we observed, however, in the left ventricle, this was not evident, possibly because LVM is higher and requires a longer time to develop loss of muscle mass as part of systemic muscle wasting.

The possible mechanism by which RHF increases the risk of developing sarcopenia may be due to increased right atrial pressure and decreased stroke volume, leading to activation of the renin–angiotensin–aldosterone system and systemic venous congestion. This congestion causes intestinal hypoperfusion, promoting increased translocation of bacterial lipopolysaccharide, increased production of pro-inflammatory cytokines, anorexia, and malabsorption. These factors trigger activation of catabolic pathways and inhibition of anabolic muscle pathways, worsening mass and strength [32,33]. In addition, sarcopenia may be associated with alterations in both the LV and the RV; however, because the left ventricle has greater muscle mass, it may require more time to develop echocardiographic changes as part of systemic muscle wasting.

In animal model experiments, Akt (protein kinase B)-mediated skeletal muscle secretion of substances with cardiac protective effects has been identified, stimulating muscle and mast cell growth, accelerating fat oxidation, enhancing insulin sensitivity, and mediating anti-inflammatory effects, reducing myocardial damage [34]. This protein is secreted by skeletal muscle [35], and Akt, a protein kinase B, is recognized as a cardiac protective factor that may reduce cardiac injury [36]. Thus, pathological changes in SMM in patients with sarcopenia may diminish the protective effects of cardiac protective factors [37].

As skeletal muscle functions as a secretory organ [38], releasing myokines such as irisin and follistatin-like protein 1 [39], in sarcopenic individuals, where muscle mass and function decrease, these protective actions also diminish.

Early detection of sarcopenia enables the early application of therapeutic strategies such as exercise and nutritional interventions, which may improve exercise tolerance, muscular function, quality of life, and prognosis [40]. Exercise is an effective therapeutic strategy that has been shown to enhance muscular mass, muscular strength, and exercise tolerance.

Moreover, optimal nutrition is an effective strategy for reducing the risk of developing sarcopenia [41]. In addition, in sarcopenic subjects, nutritional interventions, such as supplementation with proteins, essential amino acids, leucine, and β-hydroxy-β-methylbutyrate, have shown positive effects on nutritional status, improving muscle mass and strength [42].

Among the limitations of this study are the moderate sample size and the inherent limitations of a cross-sectional study, such as the inability to establish causality between sarcopenia and cardiac remodeling. However, we performed multivariate models, which allowed us to adjust for possible confounding variables. Previous studies on the relationship between sarcopenia and cardiac atrophy have focused mainly on left ventricular echocardiographic parameters; however, our study assessed echocardiographic alterations in sarcopenic and non-sarcopenic subjects with RHF, showing that in RHF, in addition to LVDD, there is a decrease in RV wall thickness despite having higher pulmonary artery systolic pressure and probably RV diastolic dysfunction evidenced by RA indexed volume, an essential point given that current evidence primarily derives from studies conducted in left HF.

Furthermore, this study highlights the need for prospective studies to evaluate changes in echocardiographic parameters in subjects with muscle alterations, such as sarcopenia, and their impact on functional status and prognosis in RHF patients.

## 5. Conclusions

The prevalence of sarcopenia in RHF patients was observed to be 24.5%. An LVDD and reduction in RV wall diameter were observed in these subjects, suggesting a loss of cardiac muscle mass after adjusting for age, sex, and COPD. However, this was not evident in the cardiac left ventricle, possibly because a higher LVM required a longer time to develop as part of systemic muscle mass wasting. Future longitudinal studies are needed to clarify causality and its impact on prognosis.

## Figures and Tables

**Table 1:** Clinical characteristics of Right Heart Failure subjects according to sarcopenia.

	All	Sarcopenia	Non-Sarcopenia	*p*-Value
	n = 183	n = 45	n = 138	
Age, years	64.34 ± 13.97	74.44 ± 10.19	61. 05 ± 13.48	<0.001
Male, n (%)	110 (60.11)	33 (73.33)	77 (55.80)	0.037
Hypertension, n (%)	87 (47.80)	24 (53.33)	63 (45.99)	0.392
COPD, n (%)	66 (36.07)	23 (51.11)	43 (31.16)	0.016
Nephropathy, n (%)	24 (13.11)	7 (15.56)	17 (12.32)	0.576
Diabetes, n (%)	65 (35.52)	14 (31.11)	51 (36.96)	0.447
Asthma, n (%)	7 (3.83)	3 (6.67)	4 (2.90)	0.366
Heart Failure type				
HFrEF, n (%)	35 (19.13)	10 (22.22)	25 (18.12)	0.220
HFmrEF, n (%)	13 (7.10)	5 (11.11)	8 (5.80)	
HFpEF, n (%)	10 (5.46)	4 (8.89)	6 (4.35)	
Total proteins, gr/dL	6.88 ± 0.69	7.02 ± 0.88	6.84 ± 0.61	0.413
Albumin, gr/dL	3.90 ± 0.38	3.79 ± 0.44	3.93 ± 0.35	0.086
Serum creatinine, mg/dL	0.9 [0.7–1.08]	0.92 [0.69–1.24]	0.88 [0.72–1.05]	0.450
Sodium, mmol/L	138.30 ± 3.30	138.38 ± 3.33	138.27 ±3.31	0.880
Potassium, mmol/L	4.26 ±0.50	4.32 ± 0.57	4.24 ± 0.48	0.520

COPD—Chronic obstructive pulmonary disease; HFrEF—Heart failure reduced ejection fraction; HFmrEF—Heart failure mid-range ejection fraction; HFpEF—Heart failure preserved ejection fraction.

**Table 2:** Echocardiographic parameters according to sarcopenia status in RHF subjects.

	All	Sarcopenia	Non-Sarcopenia	*p*-Value
	n = 183	n = 45	n = 138	
LV diastolic diameter, cm	4.52 ± 0.87	4.44 ± 0.99	4.55 ± 0.83	0.477
LV systolic diameter, cm	3.21 ± 1.23	3.38 ± 1.72	3.15 ± 1.03	0.410
Interventricular septum, cm	1.11 [1–1.3]	1.14 [0.99–1.28]	1.11 [1–1.3]	0.429
LV Posterior wall, cm	1.09 ± 0.20	1.09 ± 0.20	1.09 ± 0.20	0.908
LVTDV, mL	69.4 [50–107]	69.4 [53.3–110]	69.7 [49.18–106]	0.526
LVTSV, mL	34.8 [21.4–68.3]	55.3 [29.6–74]	33 [20.7–60]	0.053
LVMI, gr/m^2^	99.87 [76.73–118.70]	101.78 [81.84–131.43]	98.44 [72.12–117.49]	0.304
Relative Wall Thickness, mm	0.50 ± 0.14	0.52 ± 0.14	0.50 ± 0.14	0.409
LVEF, %	53.10 ± 15.82	49.40 ± 15.21	54.29 ± 15.89	0.102
Cardiac output, L/min	4.04 [3.07–5.37]	3.71 [2.61–4.71]	4.05 [3.21–5.39]	0.083
Cardiac index, L/min/m^2^	2.24 [1.63–2.90]	2.25 [1.64–2.90]	2.23 [1.60–2.90]	0.927
Left atrium vol, mL	49.42 [37–79]	50.2 [33.5–78]	49.42 [37.3–79]	0.769
Left atrium vol index, mL/m^2^	27.24 [18.83–41.99]	31.89 [17.67–47.02]	26.43 [19.42–40.05]	0.384
Basal RV Diameter, cm	3.97 ± 0.89	4.12 ± 0.97	3.92 ± 0.85	0.191
Mid-cavity RV diameter, cm	2.76 ± 0.72	2.93 ± 0.77	2.71 ± 0.70	0.098
RV Longitudinal diameter, cm	7.35 ± 1.23	7.24 ± 1.30	7.39 ± 1.21	0.491
RV thickness wall, mm	5 [4–6.87]	4 [3.81–5.15]	5.73 [4.95–7]	0.002
TAPSE, mm	17 [16–20]	17 [16.5–20]	17 [15–20]	0.512
Tricuspid S wave, cm/s	9.5 [8.75–10.3]	9.5 [8.9–10]	9.5 [8.7–10.4]	0.933
Fractional area change, %	36.68 ± 9.22	37.25 ± 10.11	36.51 ± 8.96	0.649
TAPSE/PSAP, mm/mmHg	0.48 ± 0.23	0.46 ± 0.21	0.49 ± 0.24	0.522
RA Indexed volume, mL/m^2^	24.13 [15.99–36.66]	27.12 [18.19–44.29]	22.19 [15.18–35.98]	0.090
RA pressure, mmHg	5 [5–10]	5 [5–10]	5 [5–10]	0.930
E-wave velocity, cm/s	57 [45.3–73.5]	47 [35–64.5]	59.7 [49–76]	0.006
E/e’ ratio	8.70 [6.15–12.22]	8.36 [6.44–12.39]	8.70 [5.9–12.13]	0.865
A-wave velocity, cm/s	71.84 ± 20.82	73.51 ± 20.05	71.31 ± 21.11	0.588
E/A ratio	0.74 [0.61–0.95]	0.60 [ 0.51–0.87]	0.76 [0.63–0.97]	0.005
Mitral lateral e’velocity, cm/s	7.80 ± 3.08	6.53 ± 2.88	8.22 ± 3.04	0.003
Mitral medial e’velocity, cm/s	5.28 [4–7]	4.68 [3.8–5.87]	5.55 [4–7.62]	0.022
Peak TR velocity, cm/s	2.77 [2.39–3.42]	2.98 [2.52–3.66]	2.76 [2.3–3.35]	0.072
Peak TR gradient, mmHg	31.02 [22.84–46.78]	35.52 [25.40–53.79]	30.58 [21.16–44.89]	0.074
PASP, mmHg	37.04 [29.50–54.25]	41.80 [31.16–55.12]	35.80 [28.23–51.47]	0.182

LV—Left ventricular; LVTDV—Left ventricle telediastolic volume; LVTSV—Left ventricular telesystolic volume; LVMI—Left ventricle mass index; LVEF—Left ventricular ejection fraction. RV—Right ventricle; TAPSE—Tricuspid annular plane systolic excursion; TAPSE/PSAP—Tricuspid annular plane systolic excursion to Pulmonary systolic artery pressure ratio; RA—Right atrium; PASP—Pulmonary artery systolic pressure. TR—Tricuspid regurgitation.

**Table 3:** Association between echocardiography parameters and sarcopenia in RHF subjects.

	Crude Model			Multivariate Model		
	β	95% CI	*p*-Value	β	95% CI	*p*-Value
LV diastolic diameter, cm	−0.10	−0.40 to 0.19	0.478	−0.10	−0.42 to 0.20	0.507
LV systolic diameter, cm	0.22	−0.19 to 0.64	0.289	0.23	−0.21 to 0.67	0.303
Interventricular septum, cm	−0.06	−0.14 to 0.02	0.180	−0.07	−0.16 to 0.02	0.125
LV posterior wall, cm	−0.004	−0.07 to 0.06	0.909	−0.009	−0.08 to 0.06	0.815
LVTDV, mL	4.95	−9.79 to 19.71	0.508	9.65	−5.72 to 25.03	0.217
LVTSV, mL	9.44	−9.18 to 28.08	0.317	3.38	−16.49 to 23.27	0.736
LVMI, gr/m^2^	6.44	−8.28 to 21.18	0.389	3.91	−11.60 to 19.43	0.619
Relative wall thickness, mm	0.020	−0.02 to 0.07	0.411	0.01	−0.03 to 0.07	0.504
LVEF, %	−4.88	−10.76 to 0.98	0.102	−5.33	−11.35 to 0.68	0.082
Cardiac output, L/min	−1.06	−4.36 to 2.22	0.521	−1.28	−4.73 to 2.16	0.462
Cardiac index, L/min/m^2^	−0.08	−1.96 to 1.79	0.929	−0.20	−2.16 to 1.75	0.837
Left atrium volume, mL	−3.54	−15.91 to 8.82	0.572	−0.90	−13.96 to 12.14	0.891
Left atrium volume index, mL/m^2^	3.41	−4.14 to 10.96	0.374	4.27	−3.70 to 12.25	0.292
Basal RV diameter, cm	0.20	−0.10 to 0.50	0.191	0.08	−0.23 to 0.39	0.614
Mid-cavity RV diameter, cm	0.21	−0.04 to 0.47	0.099	0.24	−0.02 to 0.52	0.076
RV longitudinal diameter, cm	−0.15	−0.59 to 0.28	0.491	−0.27	−0.69 to 0.14	0.199
RV thickness wall, cm	−1.18	−2.03 to −0.33	0.007	−1.36	−2.30 to −0.42	0.005
TAPSE, mm	1.30	−0.37 to 2.99	0.127	1.25	−0.44 to 2.94	0.147
Tricuspid S wave, cm/s	0.09	−0.63 to 0.82	0.790	−0.06	−0.83 to 0.71	0.873
Fractional area change, %	0.74	−2.48 to 3.97	0.649	1.009	−2.35 to 4.37	0.555
TAPSE/PSAP, mm/mmHg	−0.03	−0.12 to 0.06	0.522	−0.007	−0.11 to 0.09	0.887
RA Indexed volume, mL/m^2^	9.01	−0.93 to 17.09	0.029	8.57	−0.04 to 17.20	0.051
RA pressure, mmHg	0.34	−1.1 to 1.86	0.653	−0.17	−1.84 to 1.48	0.831
E-wave velocity, cm/s	−7.43	−16.07 to 1.20	0.091	−4.65	−13.62 to 4.32	0.307
E/e’ ratio	1.33	−1.76 to 4.44	0.395	2.18	−1.02 to 5.39	0.181
A-wave velocity, cm/s	2.19	−5.80 to 10.19	0.588	0.38	−7.45 to 8.22	0.923
E/A ratio	−0.24	−0.48 to −0.01	0.039	−0.14	−0.37 to 0.09	0.244
Mitral lateral e’velocity, cm/s	−1.69	−2.81 to −0.56	0.004	−1.78	−2.97 to −0.60	0.003
Mitral medial e’velocity, cm/s	−1.05	−1.97 to −0.13	0.026	−1.006	−1.99 to −0.02	0.045
Peak TR velocity, cm/s	0.22	−0.03 to 0.49	0.090	0.16	−0.12 to 0.44	0.263
Peak TR gradient, mmHg	5.71	−1.01 to 12.44	0.096	5.98	−1.67 to 13.63	0.125
PASP, mmHg	5.45	−2.17 to 13.08	0.159	1.71	−6.63 to 10.07	0.685

LV—Left ventricle; LVTDV—Left ventricle telediastolic volume; LVTSV—Left ventricular telesystolic volume; LVMI—Left ventricle mass index; LVEF—Left ventricular ejection fraction; RV—Right ventricle; TAPSE—Tricuspid annular plane systolic Excursion; TAPSE/PSAP—Tricuspid annular plane systolic excursion to Pulmonary systolic artery pressure ratio; RA—Right atrium. TR—Tricuspid regurgitation. The multivariate model was adjusted for age, sex, and COPD.

## Data Availability

The data presented in this study are available on request from the corresponding author due to restrictions of privacy, legal, and ethical reasons.

## Abbreviations

The following abbreviations are used in this manuscript:

ASMMI	Appendicular skeletal muscle mass index.
CHF	Chronic heart failure.
COPD	Chronic obstructive pulmonary disease.
HF	Heart failure.
HFpEF	Heart failure preserved ejection fraction.
HFrEF	Heart failure reduced ejection fraction.
IVSD	Interventricular septum diastolic thickness.
LVD	Left ventricle diameter.
LVDD	Left ventricular diastolic dysfunction.
LVEF	Left ventricular ejection fraction.
LVM	Left ventricular mass.
LVPWD	Left ventricular posterior wall diastolic thickness.
RV	Right ventricle.
RVD	Right ventricle diameter.
SMM	Skeletal muscle mass.
RHF	Right heart failure.
